# Synthesis of Silver Nanoparticles by Leaf Extract of *Cucumis melo* L. and Their In Vitro Antidiabetic and Anticoccidial Activities

**DOI:** 10.3390/molecules28134995

**Published:** 2023-06-26

**Authors:** Pushpa Rani, Naveen Kumar, Kantharaj Perinmbam, Sandhanasamy Devanesan, Mohamad S. AlSalhi, Nassar Asemi, Marcello Nicoletti

**Affiliations:** 1 Department of Advanced Zoology and Biotechnology, Loyola College, Chennai 600034, Tamil Nadu, India; naveenkumarc96@gmail.com; 2 PG and Research Department of Botany, Government Arts College for Men (Autonomous), Affiliated to University of Madras, Nandanam, Chennai 600035, Tamil Nadu, India; drperinbam73@gmail.com; 3 Department of Physics and Astronomy, College of Science, King Saud University, P.O. Box 2455, Riyadh 11451, Saudi Arabia; dsandhansamy@ksu.edu.sa (S.D.); nassarasemi@gmail.com (N.A.); 4 Department of Environmental Biology, Sapienza University of Rome, 00185 Rome, Italy; marcello.nicoletti@uniroma1.it

**Keywords:** silver nanoparticles, *Cucumis melo*, diabetes, coccidiosis, α-amylase, α-glucosidase, oocysts inhibition

## Abstract

In this study, silver nanoparticles were synthesized using *Cucumis melo* L. leaf extract via a green synthesis approach and their potential against diabetes and coccidiosis was tested under in vitro conditions. The phytochemical components in the leaf extract reacted with silver nitrate in solution and yielded *C. melo*-silver nanoparticles (*Cm*-AgNPs). The synthesis of AgNPs was confirmed via UV–visible spectroscopy by obtaining a peak at 440 nm. The nanoparticles were characterized by their morphology, crystallinity, and the presence of functional groups. In vitro α-amylase and α-glucosidase inhibition assays were carried out at different concentrations in the range of 20 to 100 μg/mL of *Cm*-AgNPs. The *Cm*-AgNPs exhibited enzyme inhibitory activity in a concentration-dependent manner. As the concentration of *Cm*-AgNPs increased the inhibitory activities were also increased linearly and the highest inhibition was observed at 100 μg/mL. The effectiveness of *Cm*-AgNPs against *Eimeria tenalla* was assessed by an in vitro 3-(4,5-dimethylthiazolyl-2)-2,5-diphenyltetrazolium bromide (MTT) assay using Madin–Darby bovine kidney (MDBK) cell lines. The results revealed that the viability of the oocysts and further sporulation were decreased with the increased concentration of *Cm*-AgNPs. The AgNPs synthesized from the *C. melo* leaf extract have shown promising potential against diabetes and coccidiosis, and they could be used in biomedical applications.

## 1. Introduction

The current innovations in the field of nanotechnology bring new dimensions to medical science, research and development, and our day-to-day life. The nanoparticles, either organic (carbon, C) or inorganic metals (silver, Ag, and gold, Au), play a significant role as biomedical agents and pharmaceutical products. Among the metallic nanoparticles, Ag-based nanoparticles (AgNPs) are small in size, ranging from 10–100 nm with specific physicochemical characteristics such as size, shape, electrical conductivity, optical density, and high reactive surface area [1]. These unique characteristics allow control over the different characteristics of drugs or other biomedical agents such as alteration in solubility and blood pool retention time, controlled release over short or long durations, environmentally triggered controlled release, or highly specific site-targeted delivery [2]. The AgNPs synthesized were proven to have potential therapeutic applications against many diseases, such as cancers [3,4], and antimicrobial, antifungal, and antioxidant effects [5,6,7,8].

Type 2 diabetes mellitus is one of the most commonly prevalent non-communicable and life-threatening diseases among populations around the world [1]. In normal conditions, intestinal enzymes, such as α-amylase and α-glucosidase, degrade the starch and oligosaccharides found in food to glucose, and the insulin hormone secreted by the pancreas helps glucose enter into cells to be used for body energy [9,10]. Sometimes the pancreas does not make enough insulin or the body does not use the secreted insulin properly. Thus, glucose stays in the blood and, over time, it causes the development of chronic hyperglycemia. The combination of insulin resistance and the inhibition of insulin secretion results in type 2 diabetic conditions, influenced by genetic determinants, the over-intake of food, a sedentary lifestyle, and aging [10]. Clinical studies have shown that currently available treatments like insulin injection and antihyperglycemic drugs can cause several side effects in patients when the drugs are used in the long term and slow down therapeutic responses [11]. Today, the role of biosynthesized nanoparticles is attracting the interest of the pharmaceutical industry to create safe antidiabetic drugs to be used among patients.

Coccidiosis disease is caused by the protozoan parasites of the genus Eimeria developing within the intestine of most domestic and wild animals and birds. Live attenuated and non-attenuated vaccines are available to curtail the risks of infection, but they are not cost-effective. Another disadvantage is that live vaccines need host cells to replicate and to instigate active immunity, which causes them to result in subclinical coccidiosis [12]. The control of coccidiosis is difficult due to the emergence of drug resistance and the limitations of available anticoccidial vaccines lead to frequent disease outbreaks. Therefore, alternative approaches, such as the use of AgNPs and plant extracts as anticoccidial agents are being considered and are now the focus of many researchers. The infection of chicken intestines begins with the ingestion of sporulated oocysts releasing sporocysts, which in turn release sporozoites. The discharge of sporozoites from infected oocysts/sporocysts, otherwise known as the excystation process, is a critical step in the intracellular development of parasites inside the host cells [13].

Generally, AgNPs are prepared by employing different chemical reactions, such as the reduction in Ag ions in aqueous solutions with or without stabilizing agents [14], thermal decomposition of inorganic solvents [15], chemical reduction and photoreduction in reverse-micelles processes [16,17], and microwave-assisted irradiation [18]. However, these chemical methods have certain disadvantages, such as the involvement of toxic and hazardous chemicals during the processes, being economically not viable, being unsafe to the environment, and the resultant product being biologically unsafe or incompatible with living entities [19,20]. Thus, there is a growing need to develop nontoxic, biocompatible, eco-friendly, and efficient methods to synthesize AgNPs for their use in medical and pharmaceutical products. This has caused researchers turn their focus to living organisms.

In recent times, many studies have been conducted by exploring the different kinds of organisms, such as bacteria [21], fungi [22], algae [23], and plants [24,25], to create cost-effective, safe, and environmentally compatible AgNPs. Many plants and plant extracts have been reported for the successful production of stable nanoparticles, particularly *Cantharanthus roseus* leaf extracts [26] and *Clitorea ternatea* and *Solanum nigrum* [27]. Kesharwani et al. fabricated high-density stable nanoparticles of size 16–40 nm using the leaf extracts of *Datura metal* [28]. Black tea leaf extracts were also used in the formation of AgNPs [29]. It is assumed that the leaf extracts contain a large number of active metabolites, such as alcoholic components, alkaloids, proteins, amino acids, and enzymes that aided in the reduction in Ag ions and led to the formation of AgNPs [28].

*Cucumis melo* L. is commonly known as musk melon, and it belongs to the family of Cucurbitaceae. It is a climbing annual herb. It grows well in a well-drained deep soil and it has long been cultivated mainly for its edible fruits; however, it is found as a weed in cultivated fields, natural open areas, grasslands, and wastelands [30]. *Cucumis melo* leaves are simple and alternate, approximately rounded and pubescent with cordate bases on long petioles, and with 3 to 7 shallow or irregular lobes [31]. The leaves have medicinal importance, owing to the presence of a group of phytoconstituents [32]. It has been found that the leaf extracts of the Cucurbitaceae family have antibacterial, antifungal, wound-healing, antiviral, antidiabetic (type 2), antidiarrheal, and anti-inflammatory activities [33,34].

The present study to synthesize AgNPs from the leaf extracts of *C. melo* has proven its excellent antidiabetic potential and anticoccidial efficacy under in vitro conditions.

## 2. Results

### 2.1. Synthesis and Characterization of Silver Nanoparticles Using C. melo

#### 2.1.1. UV–Visible Spectroscopy

**Figure 1:**
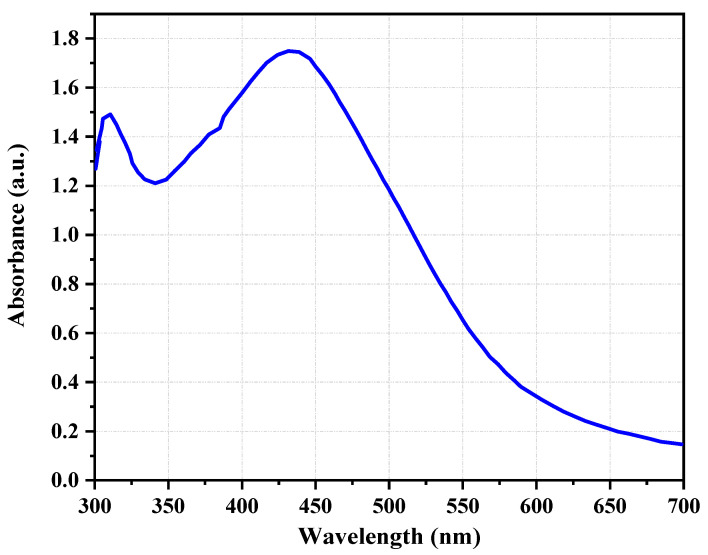
The UV–visible absorption spectrum of synthesized silver nanoparticles (AgNPs) from the leaf aqueous extract of *C. melo*.

The synthesis of AgNPs using *C. melo* leaf extract was successful, as observed visually via a shift in color from pale yellow to dark brown, and a resulting relatively stable solution in Figure 1. The color change is directly attributed to the interaction of metabolites present in the leaf extract of *C. melo* with added AgNO_3_, which leads to the reduction in Ag^+^ ions to Ag^0^ nanoparticles.

#### 2.1.2. Zeta Potential Distribution

**Figure 2:**
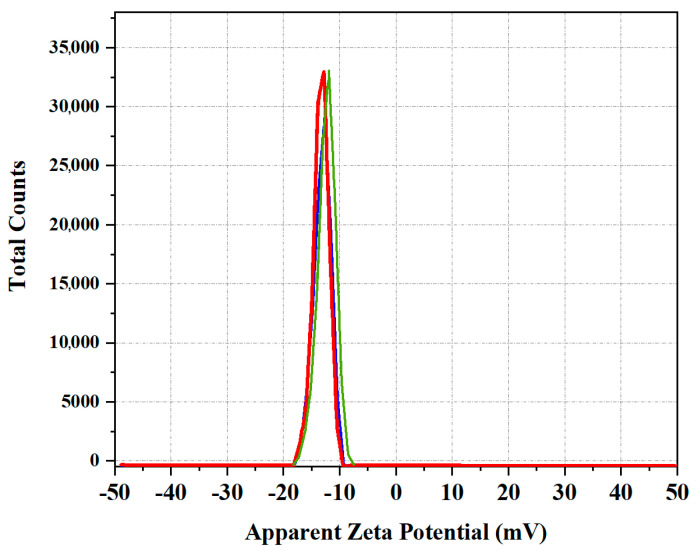
Zeta potential distribution value of synthesized *Cm*-AgNPs.

The stability of the synthesized *Cm*-AgNPs was confirmed by aqueous solution via zeta potential analysis. The zeta potential values of AgNPs were measured—12.8 mV ± 1.0 mV in Figure 2. The negatively charged surface value confirms the formation of the nanoparticles without aggregation.

#### 2.1.3. High Resolution Scanning Electron Microscopy (HR-SEM) Analysis

**Figure 3:**
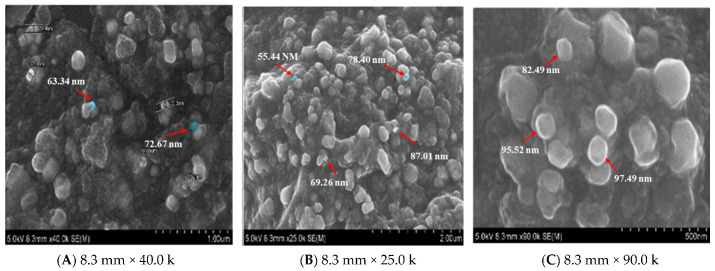
SEM images of biosynthesized silver nanoparticles (AgNPs) using *C. melo* leaf extract.

The surface morphology of biosynthesized AgNPs was analyzed using an SEM FEI-Quanta FEG 250. The surface morphology and particle size were studied from the SEM micrographs. The SEM images showed the uniform distribution of spherical shape AgNPs. The normal range of SEM observation is from 1 nm to 100 nm. The SEM micrographs of synthesized *Cm*-AgNPs magnified at 8.3 mm × 40.0 k, 8.3 mm × 25.0 k, and 8.3 mm × 90.0 k are shown in Figure 3 respectively. These different magnified images show well-defined spherical, porous, and smooth *Cm*-AgNPs. The observed range for *Cm*-AgNPs was 66.7 to 92.3 nm (Figure 3A).

#### 2.1.4. X-ray Diffraction Method

**Figure 4:**
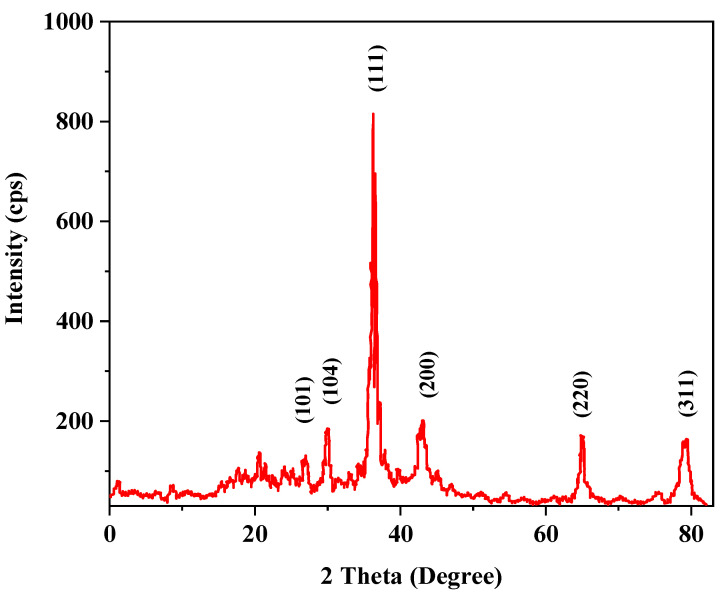
XRD pattern of *C. melo*-mediated silver nanoparticles (AgNPs).

The XRD pattern with diffraction peaks showed the crystallinity nature of silver nanoparticles. The diffraction patterns were recorded for a 2θ scan angle from 0 to 80°. The XRD pattern of biosynthesized *Cm*-AgNPs was confirmed by the characteristic peak observed in XRD image (Figure 4). In the XRD pattern, six distinct diffraction peaks were observed at 2θ scan angles of 30°, 32.7°, 40.0°, 44°, 64.2°, and 79.4°, which were indexed at (101), (104), (111), (200), (220), and (311) of the cubic and face-centered structure of metallic silver, respectively. The peaks observed in the pattern were correlated with pure silver available from the Joint Committee on Powdered Diffraction Standards (JCPDS No. 04-0783). In a comparison of biosynthesized AgNPs with the standard XRD pattern, the XRD pattern of *C. melo*-mediated AgNPs produced a high-quality nanocrystal with a different crystalline form.

#### 2.1.5. Fourier Transform Infrared (FTIR) Spectroscopy

**Figure 5:**
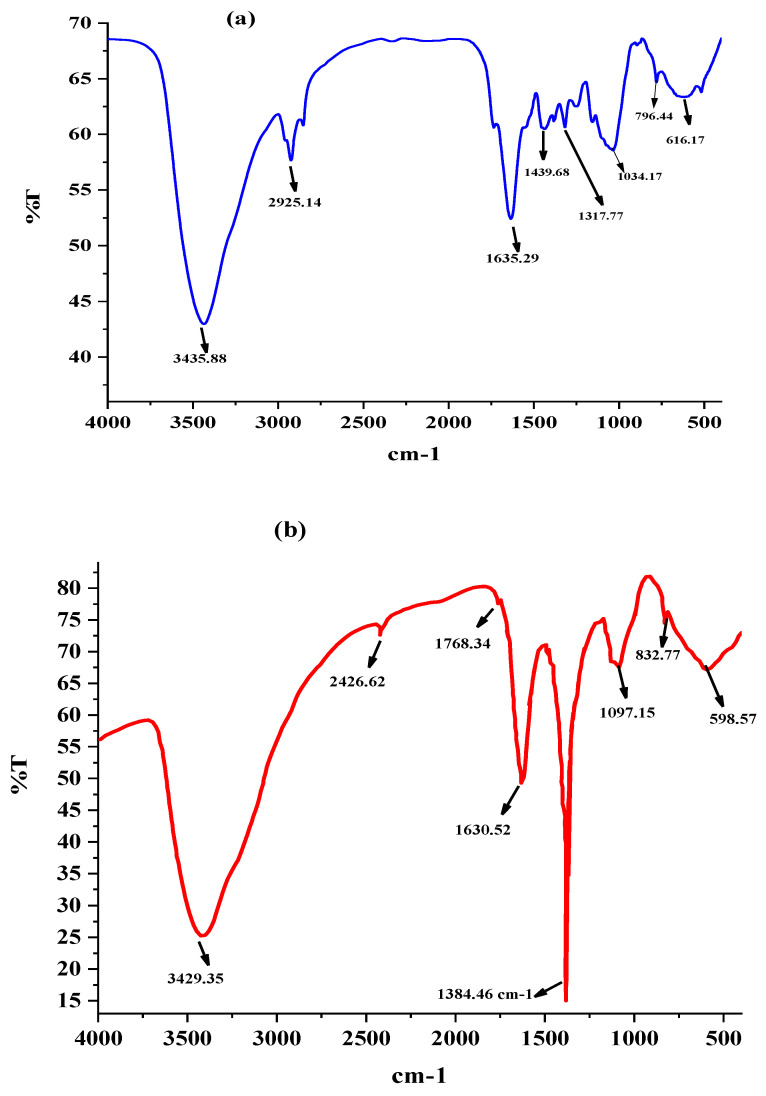
(**a**) FTIR spectrum of *C. melo* leaf extract. (**b**) FTIR spectrum of synthesized silver nanoparticles (AgNPs) using *C. melo* leaf extract.

The absorption wavelength of electromagnetic radiation is characteristic of chemical bond stretching and bending vibrations. The characteristic absorption peaks were utilized for the quantitative analysis of chemical structural elucidation, functional groups present in the nanoparticles, and the possible interaction of functional groups. The FTIR spectrum of *C. melo* leaf extract alone in Figure 5a and silver nanoparticles (*Cm*-AgNPs) in Figure 5b was recorded using KBr pellets. The infrared light passed through the pellet, and the IR the absorption spectrum of secondary metabolites involved in the reduction in silver ions and the capping of the AgNPs is shown in Figure 5b. The functional groups present in the *C. melo* leaf extract were interrupted as follows: 3435.88 cm^−1^, 2925.14 cm^−1^, 1636.29 cm^−1^, 1439.68 cm^−1^, 1317.77 cm^−1^, 1034.47 cm^−1^, 796.44 cm^−1^, and 616.17 cm^−1^. The FTIR spectrum of *Cm*-AgNPs showed major absorption peaks at 3429.35 cm^−1^, 2426.62 cm^−1^, 1768.34 cm^−1^, 1630.52 cm^−1^, 1384.48 cm^−1^, 1097.15 cm^−1^, 832.97 cm^−1^, and 598.67 cm^−1^. The FTIR spectrum indicates the presence of various phytoconstituents in the leaf extract and is also similar in Cm- AgNPs. The of *Cm*-AgNP’s spectral features are slightly shifted as compared to *C. melo* extract. It can be concluded that these phytoconstituents may be involved in the silver ion reduction and capping processes.

### 2.2. Biosynthesized Silver Nanoparticles as an Antidiabetic Agent

The antidiabetic activity of biosynthesized AgNPs from the aqueous leaf of extract of *C. melo* was assessed via α-amylase and α-Glucosidase inhibition assays. The in vitro enzyme inhibition assay was carried out with *Cm*-AgNPs at five different concentrations, namely 20, 40, 60, 80, and 100 μg/mL.

#### 2.2.1. α-Amylase Inhibitory Activity

**Figure 6:**
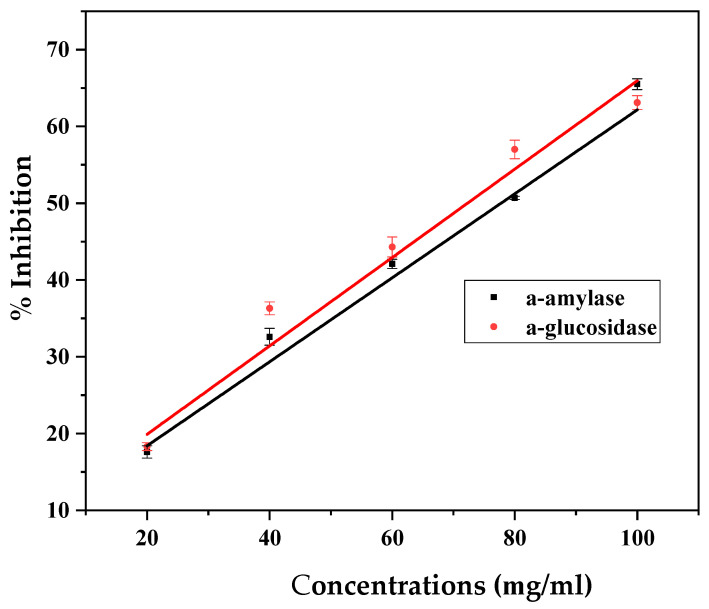
(black line) Inhibition of α-amylase and (red line) α-glucosidase activities by different concentrations of silver nanoparticles (AgNPs) synthesized from *C. melo* leaf extract.

In the present study, *Cm*-AgNPs exhibited a linear increase in amylase inhibition activity with an increase in the concentrations of nanoparticles 20 to 100 μg/mL, as shown in Figure 6 (black line). The inhibition of α-amylase was 20 μg/mL for 17.6 ± 0.8, 40 μg/mL for 32.6 ± 1.1, 60 μg/mL for 42.1 μg/mL ± 0.6, 80 μg/mL for 50.7 ± 0.2, and 100 μg/mL for 65.5 ± 0.7. The highest inhibition activity of *Cm*-AgNPs was observed at the 100 μg/mL concentration.

#### 2.2.2. Alpha-Glucosidase Inhibitory Activity

The α-glucosidase inhibitory activity of *Cm*-AgNPs is presented in Figure 6 (red line). The concentrations of *Cm*-AgNPs 20 μg/mL for 18.3 ± 0.5, 40 μg/mL for 36.3 ± 0.8, 40 μg/mL for 44.3 μg/mL ± 1.3, 80 μg/mL for 57.0. ± 1.2, and 100 μg/mL for 63.1 ± 0.9. When the concentration of *Cm*-AgNPs increased, the enzyme inhibitory effect also increased in the range of 20 to 65%. The maximum inhibitory action of *Cm*-AgNPs towards the enzyme was observed at 100 μg/mL.

### 2.3. Biosynthesized Silver Nanoparticles as Anticoccidial Agent

**Figure 7:**
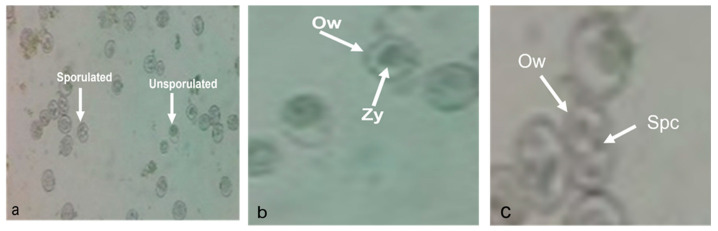
Oocysts of *E. tenella* (**a**) both unsporulated and sporulated oocysts, (**b**) enlarged unsporulated oocysts containing oocyst wall (Ow) and zygote (Zy), and (**c**) enlarged sporulated oocyst containing oocyst wall (Ow) and four sporocysts (Spc).

Initially, the fecal matter of coccidiosis-infected chickens, containing oocysts of *E. tenella*, was collected from a poultry farm. The pathological examination of infected oocysts was carried out under a confocal microscope, and the results are presented in Figure 7. The fecal matter had both unsporulated and sporulated oocysts (Figure 6 (black line)). Unsporulated oocysts of *E. tenella* were ovoid in shape with a zygote inside that was surrounded by an oocyst wall (Figure 7b), whereas the sporulated oocysts displayed an ovoid shape with four sporocysts inside that were surrounded by an oocyst wall (Figure 7c).

**Figure 8:**
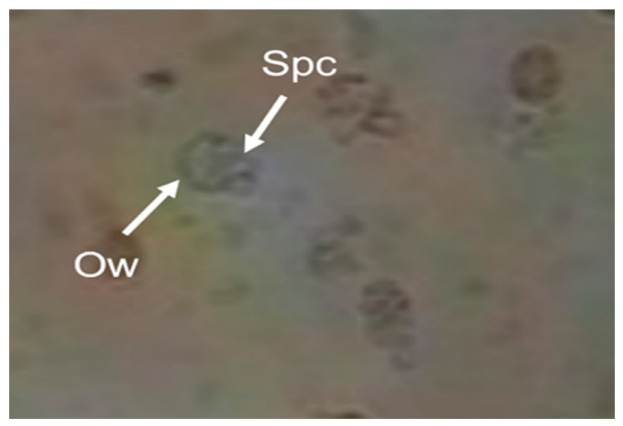
Release of sporocysts from sporulated oocysts of *E. tenella* (Ow—oocyst wall; Spc—sporocyst).

In the present study, the examination of infected oocysts under a confocal microscope revealed that the first release of sporocysts from oocysts and the excystation of sporozoites were observed after 15–20 min of incubation in PBS solution (Figure 8). Depending on the sporulated oocyst’s age and excystation solution, 60–90% of excystation occurred.

**Figure 9:**
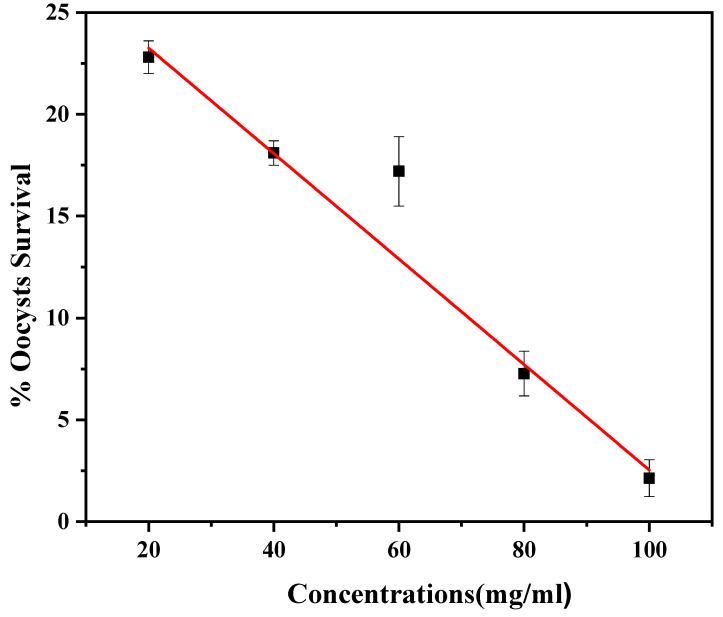
Effect of silver nanoparticles (AgNPs) synthesized from *C. melo* against *E. tenella* oocysts survival rate (%).

The effectiveness of *Cm*-AgNPs with different concentrations at 20 to 100 μg/mL against *E. tenalla* was assessed via an in vitro MTT assay using MDBK cell lines. The cell lines were used as host cells for *E. tenella*. These MDBK epithelial cells are commonly used in most in vitro studies on Eimeria. The cell lines were added with infected oocysts and synthesized *Cm*-AgNPs at different concentrations and the survival of the oocysts was monitored after 24 h of incubation in Figure 9. The results of the in vitro study demonstrated that *Cm*-AgNPs have anticoccidial potency against coccidiosis, as evidenced by cell shrinkage, and a decrease in the survival rate of oocysts as the concentrations of *Cm*-AgNPs increase. The survival rate of oocysts was evaluated using different concentrations, such as 20 μg/mL for 22.8 ± 0.8, 40 μg/mL for 18.1 ± 0.6, 60 μg/mL for 17.2 ± 1.7, 80 μg/mL 7.3 ± 1.1., and 100 μg/mL for 2.1 ± 0.7, respectively. The highest mortality of oocysts was observed at 2% at a concentration of 100 μg/mL *Cm*-AgNPs. *Cm*-AgNPs impaired the *E.tenella* in the host cells before the oocysts were completely formed and arrested the sporulation of oocysts.

## 3. Discussion

Nanotechnology has proven to be the greatest multidisciplinary field in recent years with its potential application in environmental remediation and the agriculture, chemical, and pharmaceutical industries. Despite the usefulness of metal nanoparticles, AgNPs are more appropriate in the clinical field due to their unique characteristics, such as electrical conductivity, antibacterial activity, chemical stability, etc. [35]. The chemical synthesis of AgNPs from toxic chemicals is hazardous to the environment and not suitable for biomedical applications. Therefore, the eco-friendly synthesis of AgNPs using plants, microorganisms, and biocompatible polymers [36,37] has attracted significant interest in recent years. Gopalasatheeskumar et al. reported that the total phenolic content in the leaves was 77.82 mg/g of extract while the total flavonoid content was 30.06 mg/g of extract [32]. Even though the mechanism of the bioreduction process has not been clearly ascertained, the presence of a group of phytochemical constituents, such as glycosides, alkaloids, flavonoids, terpenoid, steroids, saponin, tannin, reducing sugars, proteins, and amino acids, in the leaf extracts of *C. melo* reduced Ag^+^ ions to Ag^0^ and they, in turn, were oxidized to other species [38]. As shown by UV–visible absorption spectra in Figure 1, the AgNPs exhibited a well-defined absorption peak at 440 nm after 24 h of the reaction period. UV–visible spectroscopy is the most commonly used technique for the structural characterization of nanoparticles. The presence of a peak clearly indicates the biosynthesized AgNPs have strong surface plasmon resonance (SPR) electrons on the surface of nanoparticles [39]. A similar UV–visible spectrum at 440 nm was revealed by Baharara and co-workers while synthesizing the AgNPs using *Salvia officinalis* leaf extract [40], *Moringa oleifera* leaf extract [41], and the leaf extracts of *Ocimum Sanctum* [42].

The zeta potential value of *Cm*-AgNPs exhibited negatively charged and equally distributed particles in the aqueous medium, and similar results have been reported for *Drosera spatulate* [3]. These *Cm*-AgNP SEM results fall under the usual size range of silver nanoparticles. There were large-sized particles observed between the nanoparticles due to agglomeration. The morphology of AgNPs from the SEM results is in correlation with the previously reported AgNPs biosynthesized from *C. melo* [43]. Less than 100 nm of silver nanoparticles with spherical shapes are highly active in terms of sustainable silver release due to their vast surface region [44]. Silver nanoparticles of smaller sizes have proven antibacterial efficacy [45], antiviral properties, and other beneficial uses [46].

The IR peaks at 1640, 1748, and 3320 cm^−1^ are characteristic of AgNPs and are in close agreement with the previously synthesized AgNPs using plant extracts [47]. The IR peak at 3429.35 cm^−1^ was assigned to alcoholic O-H or N-H groups in the phenolic compound stretching vibration. A weak absorption peak at 1768.34 cm^−1^ corresponds to aldehyde, ketone, and carboxylic acid groups. From previous reports, it was predicted that the peak at 1768.34 cm^−1^ was involved (terpenoids) in the reduction process of silver ions to silver (ref). Terpenoids might have been oxidized to carboxyl groups and could have led to IR absorption in this position. A strong peak at 1630.52 cm^−1^ is suggested to be the capping of AgNPs. The peaks at 1384.48 cm^−1^, 1097.15 cm^−1^, 832.97 cm^−1^, and 598.67 cm^−1^ assigned to NO_2_, C-OH, aliphatic chloro compounds, and aliphatic bromo compounds were present in *C. melo* extract, respectively.

Pancreatic α-amylase is the key enzyme that breaks down the complex starch and carbohydrates, i.e., oligosaccharides, to disaccharides in the intestine. Hence, the inhibition activity of α-amylase offers an effective strategy to reduce the level of post-prandial hyperglycemia. Amylase inhibitors or starch blockers contain substances that prevent starch dietary components from being absorbed by the body. Therefore, the increase in blood sugar levels can be reduce through carbohydrate consumption [48]. Both *C. melo* leaf extracts [32] and biosynthesized AgNPs [48,49] have been reported as potential α-amylase inhibitors. The main role of α-glucosidase inhibitors is to inhibit the absorption of carbohydrates from the small intestine. α-glucosidase inhibitors are compared favorably to inhibitor enzymes that change complex non-absorbable into simple absorbable carbohydrates. These enzymes involved slow carbohydrate absorption and lead to a reduction in blood glucose level [50,51]. Similar dose-dependent amylase inhibition activity by *Cassia auriculata*-AgNPs was also reported by Thirumal and Sivakumar [48]. The results obtained in this study are in agreement with those of Kazeem et al., who mentioned that mild amylase inhibition is desirable compared to the excessive inhibition of amylase activity, since it leads to the abnormal bacterial fermentation of undigested starch in the colon [52]. They also inferred that the different biological components in the leaf extract might have greatly contributed to the inhibition of enzyme activity by competing with the substrate for binding to the active sites of α-amylase.

Our results closely match with the concentration-dependent inhibitory activity of biosynthesized AgNPS varied between 18.3% and 63.1% [48]. Kazeem et al. found that the phytochemicals present in leaves do not compete with the substrate for binding to the active sites on α-glucosidase; however, they compete for separate sites on enzymes and retard the cleavage of di- to monosaccharides and reduce the glucose level in the blood [52]. In line with previous reports [48,49], the AgNPs synthesized from the leaves of *C. melo* have also been shown to have strong intestinal α-glucosidase enzyme activity, which can effectively and safely be used against postprandial hyperglycemia.

Similar pathological observations were made by Kasem et al. [53]. Chickens can be infected with sporulated oocysts found in feed material, and these oocysts are the primary cause of coccidiosis spreading among chickens [53,54]. Additionally, *E. tenella* oocysts can remain viable and active for a period of nine months in poultry litter, making it a source for other poultry farms [55].

Many plant extracts-based nanoparticles have proven anticoccidial ability against Eimeria. Kasem et al. observed in vitro sporulation disruption with the chitosan-based nanoparticles from the ethanolic leaves extract of *Rosmarinus officinalis* in the oocysts of *E.tenella* [53]. Dkhil et al. reported that Ag-nanoparticles synthesized from the rhizome extracts of *Zingiber officinale* have anticoccidial ability against *E. papillata* [56]. Ismail et al. demonstrated that the leaves of *C. melo* are rich in phenolic compounds and exhibited high antioxidant potential [57]. Hence, it could be concluded that the secondary metabolites in the leaves of *C. melo* might have caused oxygen stress and disrupted the survival of the oocysts of *E. tenella*. Additionally, as suggested by Cedric et al., in extracts of *Psidium guajava*, the leaf extract might have penetrated the cell wall of oocysts and damaged the intracellular components [58].

## 4. Materials and Methods

### 4.1. Chemicals and Materials

Chemicals used in this study include silver nitrate (AgNO_3_), sodium phosphate buffer, α- amylase solution, dinitro salicylic acid, α-glucosidase solution, paranitrophenyl α-D-glucopyranoside, sodium carbonate (Na_2_CO_3_), phosphate-buffered saline (PBS) Solution, sodium hypochlorite, thiosulphate, sodium taurocholate, bovine trypsin, and Alsever’s solution. Instruments used in this study include a UV–visible spectrophotometer, an optical microscope, a confocal microscope, a scanning electron microscope, X-ray diffraction (XRD) analysis, and Fourier transform infrared (FTIR).

### 4.2. Synthesis and Characterization of Silver Nanoparticles

The fresh leaves of *C. melo* were collected from the Pallikaranai Wetland Region, Tamil Nadu, India. The collected samples were transferred to the laboratory and rinsed with distilled water before further processing to eliminate contamination. Then, the leaves were well dried under shade at room temperature (25 to 30 °C) and were pulverized into a fine coarse powder using a blender. Two grams of powdered leaves were mixed with 50 mL of distilled water and kept in a water bath with a temperature ranging from between 55 and 60 °C for 20 min. Then, the extract was filtered through Whatman No 1 filter paper and the filtrate was stored at 4 °C until further use.

**Scheme 1:**
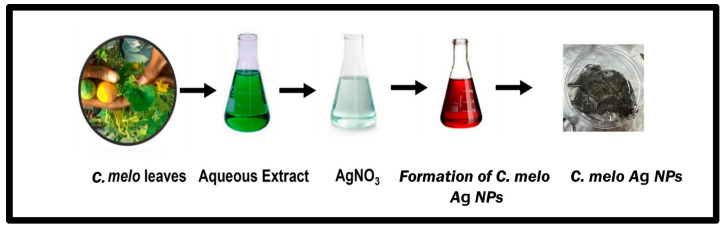
A total of 1 mL of aqueous leaf extract of *C. melo* was added to 9 mL of 1 mM silver nitrate (AgNO_3_) for AgNP synthesis. The mixture was then incubated in the dark for 24 h to achieve the bioreduction process at room temperature. The optimum condition for the formation of the silver nanoparticles was observed at near pH 7, which is a neutral condition.

All the synthesis process was carried out in the biosafety cabinet with a standard laboratory condition Scheme 1. The synthesized AgNPs from the leaf extract of *C. melo* were further characterized by their size, optical density, and morphology using different techniques, such as UV–visible spectrophotometry, SEM (JSM-6380LA (Tokyo, Japan), FTIR (Shimadzu Corporation, Kyoto, Japan), XRD (Ultima IV, Rigaku, Akishima, Japan), and Zeta Sizer (Malvern Instruments, Malvern, England).

### 4.3. Antidiabetic Screening

#### 4.3.1. α-Amylase Inhibition Assay

The α-amylase inhibition assay was performed using the DNSA method described elsewhere [59]. The assay mixture, which consisted of 500 μL of 0.02 M sodium phosphate buffer (containing 6 mM of NaCl, pH 6.9) and α-amylase solution (1 U/mL), was added with different concentrations of AgNPs (20–100 μg/mL). The assay mixture was pre-incubated at 37 °C for 20 min. After incubation, 250 μL of 1% starch solution in the aforementioned buffer was added to the tubes and incubated for 15 min at 37 °C. Finally, the catalytic reaction was terminated by adding 1 mL of di-nitrosalicylic acid reagent and then incubated in a boiling water bath (90 ± 5 °C) for 10 min. The tubes were cooled to room temperature and the absorbance was measured at 540 nm using a UV–visible spectrophotometer. The reference sample included all other reagents and enzymes except the test sample. The α-amylase inhibitory activity was expressed as a percentage inhibition, and it was calculated according to the equation below:

$$\%\;Inhibition=\frac{\left(Concentration\;of\;Sample-Concentration\;of\;control\right)}{Concentration\;of\;Sample}\times 100$$

#### 4.3.2. α-Glucosidase Inhibition Assay

The α-glucosidase inhibition assay was performed by following a modified method reported elsewhere [60]. The assay mixture, consisting of 150 μL of 0.1 M sodium phosphate buffer (containing 6 mM NaCl, pH 6.9) and 0.1 U of α-glucosidase, was added with different AgNP concentrations ranging from 20 to 100 μg/mL. The assay mixture was then pre-incubated at 37 °C for 10 min. Then, 50 μL of 2 mM paranitrophenyl α-D-glucopyranoside in 0.1 M sodium phosphate buffer was added to the mixture and incubated further to start the reaction. After 20 min of incubation at 37 °C, the reaction was terminated by adding 50 μL of 0.1 M Na_2_CO_3_. Then, the absorbance was measured at 405 nm using a UV–visible spectrophotometer. The tube with α-glucosidase but without AgNPs served as a control with 100% enzyme activity, and the antidiabetic drug acarbose was employed as a positive control. The percentage of the α-glucosidase inhibition activity was calculated as follows:

$$\%\;Inhibition=\frac{\left(Concentration\;of\;Sample-Concentration\;of\;control\right)}{Concentration\;of\;Sample}\times 100$$

### 4.4. In Vitro Anticoccidial Activity Screening

The fecal matter of coccidiosis-infected chickens, containing oocysts of *E. tenella*, was collected from a poultry farm in Tamil Nadu, India, and the samples were preserved at 4 °C until further use. The pathological examination of infected oocysts was carried out under a confocal microscope.

The release of sporocysts from infected oocysts was carried out by following a previously described method. The infected oocysts were washed and suspended in cold PBS (pH 7.2) at a concentration of 10^8^/3 mL. A total of 3 mL of 10.5% sodium hypochlorite was added to obtain a final concentration of 12% sodium hypochlorite. The mixture was stirred for 10 min, and the reaction was arrested by adding 1.05% thiosulphate. The suspension was centrifuged at 1500 rpm for 10 min at 4 °C. The oocysts pellets were re-suspended in cold PBS and re-centrifuged. The resuspension and centrifugation were repeated four times to achieve the complete removal of added sodium hypochlorite. The percentage of sporozoites excysting was increased by pre-incubating the oocysts in PBS for 1 h at 37 °C. Then, the sediment was mixed with pre-warmed excystation fluid consisting of 0.75% (*w*/*v* in PBS) sodium taurocholate (Aldrich) with 0.25% (*w*/*v* in PBS) bovine trypsin (Difco) and was incubated for 30–45 min at 37 °C in a warm water shaking bath. The excystation process was monitored microscopically.

### 4.5. An In Vitro MTT Assay on Anticoccidial Efficacy

The Madin–Darby bovine kidney (MDBK) cell lines were purchased from National Repository for cell lines in India. The (3-(4,5-dimethylthiazolyl-2)-2,5-diphenyltetrazolium bromide) assay (MTT) was performed for anticoccidial activity on MDBK cell lines [61]. The in vitro assay was carried out by using a 12-well microtiter plate. Initially, MDBK cells (100 μL) were seeded in five wells and then *C. melo* aqueous extract was added at five different doses (20–100 μL). After that, 25% of infected sporulated oocysts in 2 mL of poultry fecal matter was inoculated in all five wells. The MDBK cell culture exposed to infection was incubated for 24 h. Then, the microtiter plate was checked for oocyst survival under a confocal microscope. A reference assay, consisting of uninfected MDBK cells, was also maintained throughout the experiment. The survival rate percentage of sporulated oocysts was calculated as follows:

$$\%\;Sporozoites\;survival=\frac{\left(Concentration\;of\;the\;Sample-Control\;Sample\right)}{Concentration\;of\;the\;Sample}\times 100$$

## 5. Conclusions

In summary, we synthesized *Cucumis melo* leaf extract-mediated silver nanoparticles (*Cm*-AgNPs), which are important in clinical and therapeutic medication. Zeta potential *Cm*-AgNPs exhibited high stability with high negative zeta potential (−12.8 mV). The smaller size (less than 100 nm) of the biosynthesized *Cm*-AgNPs shows that more than 65% of α-amylase activity was inhibited at the concentration level of 100 μg/mL. The significant inhibition of α-glucosidase activity by *Cm*-AgNPs was also exhibited. The anticoccidial activity of *Cm*-AgNPs demonstrated the reduction in the survival of oocysts of *E. tenella*. However, the *Cm*-AgNPs proved to be efficient against diabetes and coccidiosis, and more in vivo work must be done for clinical field applications.

## Data Availability

The corresponding author will provide the data on request.

## Sample Availability

On request.
